# Neonatal SARS-CoV-2 positivity and short-term outcomes

**DOI:** 10.1186/s12884-026-09802-x

**Published:** 2026-08-11

**Authors:** Letícia Veríssimo Dutra, Giovana Leite Gonçalves, Mariliza Henrique Silva, Cibele Wolf Lebrão, Fernando Luiz Affonso‑Fonseca, Fabíola Isabel Suano‑Souza

**Affiliations:** 1 Paediatrics and Applied Sciences Programme at EPM-UNIFESP, Rua Botucatu 598, São Paulo, SP Brazil; 2 FMABC University Health Centre, Av. Lauro Gomes 2000, Santo André, SP Brazil; 3 Infectious Disease Specialist at São Bernardo do Campo Women’s Hospital - HM-SBC. Jardim Nova Petrópolis, São Bernardo do Campo, SP Brazil; 4 Neonatology Service at the São Bernardo do Campo Women’s Hospital - HM-SBC, Jardim Nova Petrópolis, São Bernardo do Campo, SP Brazil; 5 FMABC University Centre and UNIFESP-Diadema, Av. Lauro Gomes 2000, Santo André, SP Brazil; 6 Department of Paediatrics, EPM-UNIFESP, Centro Universitário FMABC, Rua Botucatu 598, Vila Clementino , São Paulo, SP CEP: 04023-062 Brazil

**Keywords:** COVID-19, SARS-CoV-2, Vertical transmission of infectious diseases, Newborns, Pregnant women

## Abstract

**Background:**

Neonatal SARS-CoV-2 positivity among newborns born to infected mothers appears to be uncommon, and factors associated with neonatal positivity and short-term outcomes remain incompletely understood.

**Objective:**

This study aimed to compare short-term outcomes between SARS-CoV-2-positive and SARS-CoV-2-negative newborns born to mothers with COVID-19 during pregnancy.

**Methods:**

In this matched case–control study, 263 pregnant women with COVID-19 were identified during the study period, and 218 newborns underwent RT-PCR testing for SARS-CoV-2 within the first 48 h of life. Among them, 23 newborns tested positive and were matched (1:2) with 46 newborns who tested negative according to clinically relevant maternal and neonatal characteristics, including the interval between maternal infection and delivery, maternal disease severity, gestational age, newborn sex, and birth weight appropriateness for gestational age.

**Results:**

The mean gestational age and birth weight of the newborns were 38.2 ± 1.7 weeks and 2994.2 ± 464.5 g, respectively. Fifteen newborns (21.7%) experienced complications during hospitalization, most commonly hypotonia/hypoactivity (13.0%) and early respiratory distress (10.1%). Respiratory distress was associated with neonatal SARS-CoV-2 positivity (OR = 21.31; 95% CI 2.24–201.68; *p* = 0.008). In addition, maternal COVID-19 infection occurring closer to delivery was associated with an increased likelihood of neonatal SARS-CoV-2 positivity (OR = 1.007; 95% CI 1.001–1.014; *p* = 0.026).

**Conclusions:**

SARS-CoV-2-positive newborns appeared to experience respiratory complications more frequently during hospitalization, while maternal infection closer to delivery was associated with neonatal positivity. Given the observational design and relatively small sample size, these findings should be interpreted with caution.

**Supplementary Information:**

The online version contains supplementary material available at https://doi.org/10.1186/s12884-026-09802-x.

## Background

The first cases of a respiratory syndrome characterized by fever, cough, dyspnoea, and atypical pneumonia were identified in late 2019 in Wuhan, China. This event marked the beginning of the pandemic caused by severe acute respiratory syndrome coronavirus 2 (SARS-CoV-2), the etiological agent of coronavirus disease 2019 (COVID-19) [1, 2]. At the beginning of the COVID-19 pandemic, before widespread vaccination became available, Brazil experienced one of the highest global burdens of COVID-19 cases and deaths, with substantial impacts on maternal and neonatal health. The country also reported one of the highest maternal mortality rates associated with COVID-19 worldwide [3].

The primary route of SARS-CoV-2 transmission is exposure to respiratory droplets and close contact with infected individuals, particularly during the symptomatic phase. However, mother-to-child transmission remains incompletely understood. Previous studies have reported that approximately 1.9% to 5.0% of newborns born to women infected with COVID-19 during pregnancy test positive for SARS-CoV-2 within the first 24–48 h of life [4]. In 2021, the World Health Organization (WHO) recognized SARS-CoV-2 as a potentially vertically transmissible infectious agent [5]. Although in utero and intrapartum transmission appear uncommon, both routes have been documented. Likewise, transmission through human milk remains unconfirmed, as SARS-CoV-2 RNA is rarely detected in milk samples and, when present, usually occurs at very low viral loads without confirmed viral replication [6].

Newborns born to mothers with COVID-19 during pregnancy may present less favourable perinatal outcomes, including lower birth weight, increased risk of respiratory distress, prematurity, neonatal death, lower Apgar scores, and a greater likelihood of admission to neonatal intensive care units [7].

To date, factors associated with neonatal SARS-CoV-2 positivity and early neonatal outcomes remain incompletely understood. Therefore, this study aimed to compare short-term outcomes between SARS-CoV-2-positive and SARS-CoV-2-negative newborns born to mothers with COVID-19 during pregnancy and to explore factors associated with neonatal SARS-CoV-2 positivity.

## Methods

### Study design and participants

This single-center matched case–control study was conducted at the São Bernardo do Campo Women’s Hospital (HM-SBC), São Paulo, Brazil, between March 2020 and December 2021. The study included newborns born to women with confirmed COVID-19 during pregnancy.

During the study period, the municipality of São Bernardo do Campo systematically performed COVID-19 testing among pregnant women during prenatal care and at delivery when clinically indicated [8]. Newborns born to mothers with COVID-19 during pregnancy were eligible for SARS-CoV-2 RT-PCR testing within the first 48 h of life using oropharyngeal swab samples.

**Fig. 1:**
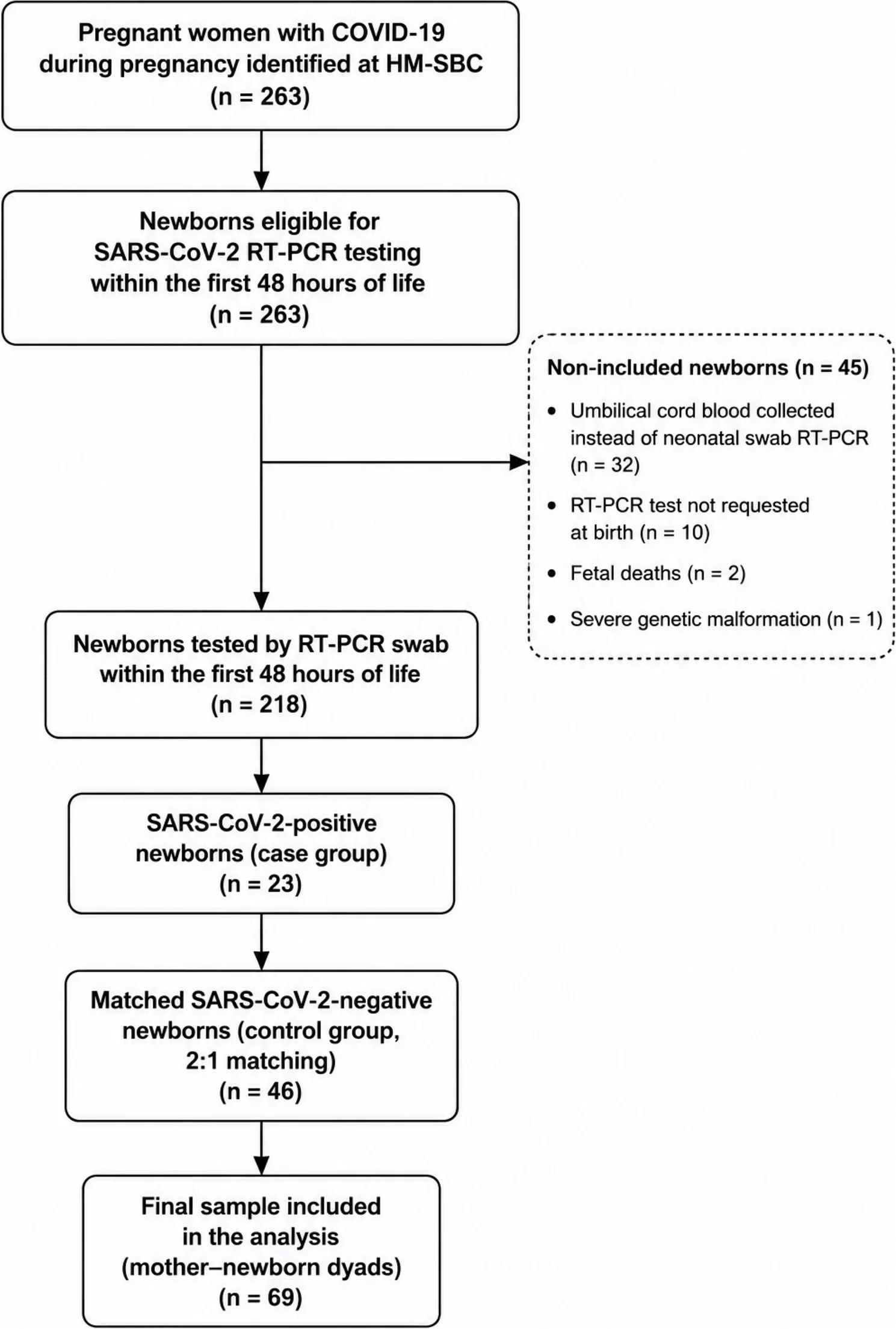
Inclusion flowchart

A total of 263 pregnant women with COVID-19 during pregnancy were identified. Of the corresponding newborns, 218 underwent RT-PCR testing within the first 48 h of life. Forty-five newborns were not included for the following reasons: umbilical cord blood was collected instead of neonatal swab RT-PCR (*n* = 32), RT-PCR testing was not requested at birth (*n* = 10), fetal death (*n* = 2), or severe genetic malformation (*n* = 1). The participant selection process is presented in Fig. 1.

Among the 218 tested newborns, 23 tested positive for SARS-CoV-2 and were included as the case group. These newborns were matched in a 1:2 ratio with 46 SARS-CoV-2-negative newborns born to mothers with COVID-19 during pregnancy (control group). Matching was performed according to clinically relevant maternal and neonatal characteristics, including the interval between maternal COVID-19 diagnosis and delivery (± 1 week), maternal disease severity, gestational age, newborn sex, and birth weight appropriateness for gestational age.

The study protocol was approved by the Research Ethics Committee of the ABC Medical School University Center (approval number 34.587.520.3.0000.0082; July 30, 2020). All procedures followed the principles of the Declaration of Helsinki and its subsequent amendments. Written informed consent was obtained from all participants after explanation of the study procedures.

### Exposure variable

The exposure variable was neonatal SARS-CoV-2 positivity, defined as a positive RT-PCR result obtained from an oropharyngeal swab collected within the first 48 h of life in newborns of women who had COVID-19 during pregnancy or delivery.

### Covariates

#### Maternal health and COVID-19 characteristics

Maternal data were collected through a structured questionnaire developed for this study (Supplementary Material), prenatal care records, and hospital medical charts. Information included socioeconomic status, maternal education, use of iron and folic acid supplementation, and maternal health conditions before and during pregnancy.

Maternal COVID-19 infection was confirmed by a positive SARS-CoV-2 RT-PCR test or by the presence of signs and symptoms suggestive of COVID-19 (including cough, dyspnea, altered taste, chest pain, altered mental status, or difficulty breathing) associated with anti-SARS-CoV-2 nucleocapsid antibody detection in serum, plasma, or whole blood [1]. Maternal disease severity was classified as asymptomatic, mild, moderate, severe, or critical according to established criteria [1]. The interval between maternal COVID-19 diagnosis and delivery was calculated in days.

#### Delivery and neonatal characteristics

Medical records were reviewed to obtain information on mode of delivery, birth conditions, neonatal resuscitation procedures, skin-to-skin contact, and breastfeeding during the first hour of life.

Gestational age was estimated based on the date of the last menstrual period, first-trimester ultrasound findings, and neonatal clinical assessment [9]. Birth anthropometric measures (weight, length, and head circumference) were recorded within the first 24 h of life. Prematurity was defined as gestational age < 37 weeks. Birth weight adequacy for gestational age was classified as small (SGA), adequate (AGA), or large for gestational age (LGA) [10].

#### Outcomes

The primary outcomes included neonatal clinical symptoms and length of hospital stay until discharge. Clinical symptoms assessed during hospitalization included fever (temperature > 37.5 °C), hypothermia (< 35.5 °C), respiratory distress syndrome (tachypnoea, subdiaphragmatic retractions, and oxygen desaturation), hypotonia, hypoactivity, hypoglycaemia (blood glucose < 40 mg/dL), cyanosis, vomiting/regurgitation, and abdominal distension.

### Statistical analysis

*Data were tabulated and analysed using SPSS version 29.0 (IBM Corp.*, Armonk, NY, USA). Continuous variables were assessed for normality using kurtosis and the Kolmogorov–Smirnov test. Parametric continuous variables were compared using Student’s t-test, whereas nonparametric variables were analysed using the Mann–Whitney U test.

Categorical variables were compared using Pearson’s chi-square test or Fisher’s exact test, as appropriate. Odds ratios (ORs) and respective 95% confidence intervals (95% CIs) were calculated for categorical outcomes.

Binary logistic regression analysis was performed to identify variables independently associated with neonatal SARS-CoV-2 positivity. Variables with *p* < 0.10 in bivariate analyses were included in the regression model. Statistical significance was set at *p* < 0.05.

## Results

A total of 69 mother–newborn dyads were included in this study. Among them, 23 newborns tested positive for SARS-CoV-2 by RT-PCR within the first 48 h of life and were matched in a 1:2 ratio with 46 newborns who tested negative. All mothers included in the study had confirmed COVID-19 during pregnancy.

The mean maternal age was 28.5 ± 6.4 years, and the median interval between maternal COVID-19 diagnosis and delivery was 86.0 days (interquartile range: 26.5–179.0 days). Twenty-one women (30.4%) had COVID-19 infection at the time of delivery. Among symptomatic pregnant women, mild disease predominated (80.7%) (Table 1).

**Table 1:** Characteristics of COVID-19 infection in pregnant women

Variables	Description	*N* (%)
COVID-19 infection at the time of delivery	Yes	21 (30.43)
Diagnosis of COVID-19 during pregnancy	Clinical/serological	40 (57.9)
	RT‒PCR	29 (42)
Classification of maternal COVID-19 infection	Asymptomatic	17 (24.6)
Severity of infection among symptomatic women	Mild	42 (80.7)
	Moderate	4 (7.7)
	Severe/Critical	6 (11.5)
Associated diseases	Asthma	2 (11.5)
	Arterial Hypertension	12 (17.4)
	Gestational Diabetes	3 (4.3)
	Hypothyroidism	2 (2.9)

*RT‒PCR* reverse transcription reaction followed by polymerase chain reaction

The mean gestational age and birth weight of the newborns were 38.2 ± 1.7 weeks and 2994.2 ± 464.5 g, respectively. Most newborns were born vaginally, at term, and with adequate weight for gestational age (Table 2). Neonatal resuscitation procedures were required in two newborns, both of whom tested positive for SARS-CoV-2 and were born to mothers diagnosed with COVID-19 at 15 and 28 weeks of gestation.

**Table 2:** General characteristics of the newborns (*n* = 69)

Variables	Description	*N* (%)
Newborn’s sex	Female	36 (52.2)
Delivery type	Caesarean	32 (46.4)
Apgar 5th minute	< 7	2 (2.9)
Resuscitation	Yes	2 (2.9)
Prematurity	Yes	11 (15.9)
Adequacy for gestational age	Small	8 (11.6)
	Adequate	58 (84.1)
	Large	3 (4.3)
Birth weight	< 2500 g	10 (14.5)
Discharge on exclusive breastfeeding	Yes	64 (92.8)
Newborn hospitalization	Intensive Care Unit	1 (1.4)
	Intermediate Care Unit	9 (13.0)
	Rooming-in	59 (85.5)
Length of stay	> 3 days	15 (21.7)
Newborn complications	Yes	15 (21.7)
Oxygen therapy	Yes	5 (7.2)
Non-invasive ventilation	Yes	3 (4.3)
CPAP	Yes	1 (1.4)
Orotracheal intubation	Yes	1 (1.4)

*CPAP* continuous positive airway pressure

Most newborns remained in rooming-in care (85.5%) and were discharged on exclusive breastfeeding (92.8%). Overall, 15 newborns (21.7%) experienced complications during hospitalization. The most frequently observed complications were hypotonia/hypoactivity (13.0%) and early respiratory distress (10.1%), whereas hypoglycaemia and vomiting/regurgitation were uncommon (1.4%) (Table 2).

A total of 11 newborns were born preterm. Among SARS-CoV-2-positive newborns, four preterm infants were identified, including one born at 35 weeks and three born at 36 weeks of gestation. Among SARS-CoV-2-negative newborns, seven preterm infants were identified, including one born at 34 weeks, one at 35 weeks, and five at 36 weeks of gestation.

In the comparison between groups, newborns who tested positive for SARS-CoV-2 appeared to experience respiratory complications more frequently during hospitalization, including early respiratory distress (26.1% vs. 2.2%; OR = 15.88, *p* = 0.004) and oxygen use (21.7% vs. 0.0%; *p* = 0.003) (Table 3). However, no significant differences were observed between SARS-CoV-2-positive and negative newborns regarding prematurity, low birth weight, adequacy for gestational age, duration of hospital stay, or hospitalization setting.

**Table 3:** Comparison of the variables studied between RT‒PCR-positive and RT‒PCR-negative newborns

Variables		Newborns				
		RT‒PCR (+) (*N* = 23)	RT‒PCR (-) (*N* = 46)	Odds ratio	95% Confidence Interval	*P* value
Newborn’s sex	Male	11 (47.8)	22 (47.8)	1.00	0.36 to 2.72	0.810
Delivery type	Caesarean	10 (43.5)	22 (47.8)	0.83	0.30 to 2.29	0.804
Gestational age	Preterm	4 (17,4)	7 (15.2)	1.17	0.30 to 4.50	0.830
Adequacy for gestational age	SGA	3 (13)	5 (10.9)	1.23	0.27 to 5.67	0.532
Birth weight	< 2500 g	3 (13)	7 (15.2)	0.83	0.19 to 3.58	0.814
Use of infant formula	Yes	2 (8.7)	3 (6.5)	1.36	0.21 to 8.80	0.746
Hospitalization location	ICU/IntCU	4 (17.4)	6 (13)	1.40	0.35 to 5.56	0.723
Length of stay	> 3 days	6 (26.1)	9 (19.6)	1.45	0.44 to 4.73	0.551
Complications	Yes	8 (34.8)	7 (15.2)	2.97	0.91 to 9.63	0.120
Early respiratory distress	Yes	6 (26.1)	1 (2.2)	15.88	1.77 to 142.8	0.004
Hypotonia	Yes	5 (21.7)	4 (8.7)	2.91	0.70 to 12.13	0.148
Oxygen use	Yes	5 (21.7)	0 (0.0)	NC	NC	0.003
COVID-19 on delivery	Yes	6 (26)	15 (32.6)	0.53	0.10 to 2.78	0.703
Maternal COVID-19	Symptomatic	18 (78.5)	34 (73.9)	1.27	0.38 to 4.17	0.777
Maternal COVID-19 (severity)	Moderate/Severe	4 (22.2)	6 (17.6)	1.33	0.32 a 5.50	0.723

*SGA* small for gestational age, *ICU* intensive care unit, *IntCU* intermediate care unit

In binary logistic regression analysis, neonatal respiratory distress remained associated with SARS-CoV-2 positivity (OR = 21.31; 95% CI 2.24–201.68; *p* = 0.008). In addition, maternal COVID-19 infection occurring closer to delivery was associated with an increased likelihood of neonatal SARS-CoV-2 positivity (OR = 1.007; 95% CI 1.001–1.014; *p* = 0.026). No association was observed between neonatal SARS-CoV-2 positivity and maternal disease severity, prematurity, or birth weight in this sample.

## Discussion

In this study, newborns who tested positive for SARS-CoV-2 within the first 48 h of life appeared to experience respiratory complications more frequently during hospitalization, particularly early respiratory distress and oxygen use. In addition, maternal COVID-19 infection occurring closer to delivery was associated with an increased likelihood of neonatal SARS-CoV-2 positivity. These findings should be interpreted cautiously given the observational design and relatively small sample size.

Mild COVID-19 predominated among pregnant women in this cohort. Extensive testing during prenatal care and access to healthcare services during pregnancy may have contributed to earlier diagnosis and timely clinical management, potentially reducing maternal complications compared with reports from settings with more limited healthcare access [11–13].

Although previous studies have reported higher rates of prematurity among pregnancies complicated by maternal COVID-19 infection, particularly in the third trimester [14], no differences in prematurity, birth weight, or adequacy for gestational age were observed between SARS-CoV-2-positive and SARS-CoV-2-negative newborns in the present study. Nevertheless, the frequency of prematurity in this cohort was slightly higher than that reported at the same centre before the pandemic [15]. Because all mothers included in this study had COVID-19 during pregnancy, these findings should not be interpreted as evidence regarding the independent effect of maternal SARS-CoV-2 infection on neonatal outcomes.

Rooming-in care and exclusive breastfeeding until hospital discharge were observed in most newborns, in agreement with World Health Organization recommendations issued during the pandemic [16, 17]. Although SARS-CoV-2 RNA has occasionally been detected in breast milk samples, viral replication and breastfeeding-mediated transmission have not been confirmed [18, 19]. Human milk may also provide passive immunological protection through immunoglobulins and other bioactive compounds, particularly secretory IgA [20].

Respiratory distress and oxygen use appeared to be more frequent among SARS-CoV-2-positive newborns in this study. Similar findings have been reported in previous studies, in which respiratory manifestations—including tachypnoea, cyanosis, and oxygen desaturation—were among the most commonly reported symptoms in neonates and infants with COVID-19 [21–24]. However, clinical presentations remain heterogeneous, and asymptomatic cases are also frequently described [25].

The mechanisms underlying neonatal SARS-CoV-2 positivity remain incompletely understood. Vertical transmission appears to be uncommon, and neonatal infection may occur in utero, intrapartum, or during the immediate postpartum period [26]. In the present study, maternal infection occurring closer to delivery was associated with a greater likelihood of neonatal positivity.

Similarly, a Brazilian cohort study reported that shorter intervals between maternal infection and delivery were associated with increased neonatal SARS-CoV-2 positivity [27]. Placental studies have also demonstrated histopathological abnormalities and the presence of viral material in syncytiotrophoblasts, supporting the biological plausibility of maternal–foetal transmission, although its clinical significance remains uncertain [28].

Two fetal deaths were observed in pregnancies in which mothers had COVID-19 at the time of delivery. Previous studies have suggested that maternal SARS-CoV-2 infection may contribute to placental dysfunction and inflammatory dysregulation, potentially affecting fetal outcomes [29, 30]. However, given the small number of events in the present study, no conclusions can be drawn regarding this association.

This study has several limitations that should be considered when interpreting the findings. First, this was a single-center study with a relatively small sample size, particularly among SARS-CoV-2-positive newborns, which limited statistical power and contributed to wide confidence intervals for some outcomes. Second, due to the observational design and inclusion of only mothers infected with COVID-19 during pregnancy, the study does not allow assessment of the independent effect of maternal SARS-CoV-2 infection compared with non-infected mothers. Third, the non-inclusion of some eligible newborns who did not undergo RT-PCR testing may have introduced selection bias, although participant selection and matching procedures were carefully described to minimize this risk.

In addition, maternal symptom data may have been subject to recall bias, and some prenatal records contained incomplete information. Data collection occurred during the early phase of the pandemic, when institutional protocols, testing strategies, and healthcare resources were evolving, which may also have influenced case identification and clinical management. Finally, neonatal follow-up was restricted to hospitalization, limiting the evaluation of longer-term outcomes.

Despite these limitations, this study has important strengths, including the matched case–control design within the same period, systematic neonatal RT-PCR testing, access to maternal COVID-19 diagnostic information during prenatal care, and detailed data regarding delivery and neonatal outcomes. These findings contribute to the growing literature on neonatal SARS-CoV-2 positivity and short-term neonatal outcomes among newborns of mothers infected during pregnancy.

## Conclusion

In this study, SARS-CoV-2-positive newborns appeared more likely to experience respiratory complications during hospitalization, while maternal infection occurring closer to delivery was associated with an increased likelihood of neonatal SARS-CoV-2 positivity. No statistically significant association was observed in this sample between neonatal positivity and maternal disease severity, prematurity, or birth weight. Given the observational design, single-center setting, and relatively small sample size, these findings should be interpreted with caution. Further studies with larger populations and longer follow-up are needed to better understand factors associated with neonatal SARS-CoV-2 positivity and its short- and long-term clinical outcomes.

## Supplementary Information

Supplementary Material 1.

## Data Availability

The data generated or analysed during this study are available upon request from the corresponding author.

## Abbreviations

SARS-CoV-2: Severe acute respiratory syndrome coronavirus 2
COVID: 19-Coronavirus Disease 2019
NB: Newborns
RT‒PCR: Reverse transcription reaction followed by polymerase chain reaction
SGA: Small for Gestational Age
AGA: Adequate for Gestational Age
LGA: Large for Gestational Age
CPAP: Continuous positive airway pressure
ICU: Intensive Care Unit
