# Supplementary material for: Neonatal SARS-CoV-2 positivity and short-term outcomes
Source: BMC Pregnancy Childbirth. 2026 Aug 11;26:1288. doi: 10.1186/s12884-026-09802-x (PMC13741664; doi:10.1186/s12884-026-09802-x)
Supplement: Supplementary file 1 [file 12884_2026_9802_MOESM1_ESM.docx]

**SURVEY**

| **Questionnaire (n°: ) Date:( / / ) Interviewer:** | | | | | | | | | |
| --- | --- | --- | --- | --- | --- | --- | --- | --- | --- |
| **IDENTIFICATION (MATERNAL)** | | | | | | | | | |
| HR/SAME: POSTCODE: | | | | | | | | | |
| Contact telephone: E-mail: | | | | | | | | | |
| Full name (mother): Date of birth: / / | | | | | | | | | |
| Full name (father): Date of birth | | | | | | | | | |
| Ethnicity: ( ) white (Caucasian) ( ) brown ( ) black ( ) indigenous ( ) oriental (yellow) | | | | | | | | | |
| Profession: | | | | | | | | | |
| Work (time): ( ) full time ( ) part time ( ) unemployed ( ) retired | | | | | | | | | |
| Schooling: __________ years of study ( ) 1 G completed ( ) 2 G completed ( ) Higher education completed | | | | | | | | | |
| Family situation : ( ) Married/partnered ( ) separated/divorced ( ) single ( ) widowed | | | | | | | | | |
| Living situation (live with): ( ) alone ( ) partner ( ) children ( ) family members ( ) other | | | | | | | | | |
| Number of people in the household : ( ) adults ( ) children | | | | | | | | | |
| Family income: R$ | | | | | | | | | |
| Surrounding conditions (housing, rubbish collection, sewage and running water) | | | | | | | | | |
| Dwelling: ( ) Brick ( ) Wood. Rubbish collection ( ) No ( ) Yes. x/week | | | | | | | | | |
| Piped water : ( ) yes ( ) no Piped sewage: ( ) yes ( ) no | | | | | | | | | |
| **MORBID, OBSTETRIC AND GESTATIONAL HISTORY** | | | | | | | | | |
| Pregnancies: Abortions: Deliveries: LMP: / / PPD: / / | | | | | | | | | |
| Start of antenatal care: (____/_____/______) Number of appointments: | | | | | | | | | |
| Place where you had your antenatal care: ( ) CAISM. ( ) UBS. Which? | | | | | | | | | |
| Nutritional status: Usual weight: kg Current weight: kg Final gestational weight: kg (Height: cm) | | | | | | | | | |
| Habits during pregnancy (medication, vitamin supplements, sun exposure and physical activity) | | | | | | | | | |
| Alcoholism: ( ) no ( ) yes. Type and dose: _________________________________________  Smoking: ( ) no ( ) yes. Cigarettes/day: _________________________________________  Illicit drugs: ( ) no ( ) yes. Which drugs? _______________________________________________  Multivitamin ( )no ( ) yes. What dose, start and end? __________________________________  Vitamin D ( )no ( ) yes. What dose, start and end? __________________________________  Folic acid ( )no ( ) yes. What dose, start and end? _________________________________  Ferrous sulphate ( )no ( ) yes. What dose, start and end? _________________________________  Sunscreen ( ) no ( ) yes. Qual frequência?_______________________________________  Antenatal corticotherapy ( )no ( ) yes. What dose, start and end? _________________________________  Other medicines ( ) no ( ) yes. Which? ______________________________________________  Sun exposure ( ) no ( ) yes. Time, frequency and duration/day? _________________________ | | | | | | | | | |
| **Birth complications** | | | | | | | | | |
| ( ) none ( ) HPP ( ) other ____________ | | | | | | | | | |
| **Obstetric USG** | | | | | | | | | |
| Date: __/___/___ changed ( ) yes ( ) no describe____________________________________  Date: __/___/___ changed ( ) yes ( ) no describe____________________________________  Date: __/___/___ changed ( ) yes ( ) no describe____________________________________  Date: __/___/___ changed ( ) yes ( ) no describe____________________________________  Date: __/___/___ changed ( ) yes ( ) no describe____________________________________  Date: __/___/___ changed ( ) yes ( ) no describe____________________________________  Date: __/___/___ changed ( ) yes ( ) no describe____________________________________ | | | | | | | | | |
| **Pregnancy complications/illnesses** | | | | | | | | | |
| Urinary infection ( ) no ( ) yes. Medication (which) ?  Other infections ( ) no ( ) yes. Medication (which) ?  Hypertension ( ) no ( ) yes. Medication (which) ?  Diabetes ( ) no ( ) yes. Medication (which) ?  Bleeding ( ) no ( ) yes. When and treatment (which) ?  Syphilis ( ) no ( ) yes. Medication (which) ?  Cytomegalovirus ( ) no ( ) yes. Medication (which) ?  Toxoplasmosis ( ) no ( ) yes. Medication (which) ?  HIV ( ) no ( ) yes. Medication (which) ?  Other. What is it? _______________________ treatment  Other. What is it? _______________________ treatment  Other. What is it? _______________________ treatment  Other. What is it? _______________________ treatment  Other. What is it? _______________________ treatment  Other. What is it? _______________________ treatment: | | | | | | | | | |
| **FLU SYNDROME** | | | | | | | | | |
| Date of onset of symptoms: / / (duration in days) | | | | | | | | | |
| Fever ( ) (days______) Dry cough ( ) (days______) Productive cough ( ) (days______) Asthenia ( ) (days______) Anorexia ( ) (days______) Myalgia (days______) Dyspnoea ( ) (days______) Nasal discharge ( ) (days______)  Conjunctival discharge ( ) (days______) Difficulty swallowing (days______) Sore throat (days______)  Runny nose ( ) (days______) Signs of cyanosis ( ) (days______) Loss of taste ( ) (days______) Diarrhoea ( ) (days______)  Loss of sense of smell ( ) (days______) Conjunctivitis ( ) (days______) Skin rashes ( ) (days______)  Others (days: ________________________________________________________________________________) | | | | | | | | | |
| **Clinical assessment** | | | | | | | | | |
| First appointment (date: _____/_____/_____): Location ( )  HR: _______ FR: _______ T: ________ BP: ________ O saturation :_2_ ________ Pain: ______________  Return 1 (date: _____/_____/_____): Location ( )  HR: _______ FR: _______ T: ________ BP: ________ O saturation :_2_ ________ Pain: ______________  Return 2 (date: _____/_____/_____): Location ( )  HR: _______ FR: _______ T: ________ BP: ________ O saturation :_2_ ________ Pain: ______________  Return 3 (date: _____/_____/_____): Location ( )  HR: _______ FR: _______ T: ________ BP: ________ O saturation :_2_ ________ Pain: _______________ | | | | | | | | | |
| Need for hospitalisation ( ) No ( ) Yes | | | | | | | | | |
| Infirmary (date: _____/_____/____). Number of days ( ).  Ventilatory support: ( ) No ( ) Oxygen catheter | | | | | | | | | |
| Intensive care unit (date: _____/_____/____). Number of days ( ) | | | | | | | | | |
| **Risk factors for worsening flu syndrome** | | | | | | | | | |
| Cardiological (arrhythmia, heart failure, pacemaker, congenital heart disease) ( ) Which: _____________________  Hypertension ( ) Medication (which)? _________________________________________________  Diabetes mellitus (type 2, gestational) ( ) Medication (which) ? ______________________________  Thyroid disease (hyperthyroidism, hypothyroidism, thyroid tube, thyroid cyst) ( ). Medications (which ones) ?  Kidney disease (chronic kidney disease, tubulopathy, nephrotic syndrome) ( ). Medication (which) ?  Vascular disease (thrombosis, coagulopathy, varicose veins, stroke) ( ). Medication (which) ?  Rheumatological disease (rheumatoid arthritis, fibromyalgia, Lupus, etc.) ( ). Medication (which) ?  Lung disease (asthma, bronchitis, malformation, COPD) ( ). Medication (which) ?  Neoplasms ( ). Medicines (which ones) ?  Obesity ( ). Medicines (which ones) ?  Others (which ones and treatment): | | | | | | | | | |
| **Place of care for flu syndrome** | | | | | | | | | |
| UBS ( ). Date first time: _____/_____/_____ How many times ?  UPA ( ). Date first time: _____/_____/_____ How many times ?  PA HM ( ). Date first time: _____/_____/_____ How many times ?  Hospitalisation in a ward. Date first time: _____/_____/_____ (n of days: ) How many times ? _________________  ICU admission. Date first time: _____/_____/_____ (n of days: ) How many times ? _______________________ | | | | | | | | | |
| **Diagnostic tests for flu syndrome** | | | | | | | | | |
| Oropharynx (RT-PCR) Date ____/_____/____ Result ____/_____/____ Positive ( ) Negative ( )  Oropharynx (RT-PCR) Date ____/_____/____ Result ____/_____/____ Positive ( ) Negative ( )  Oropharynx (RT-PCR) Date ____/_____/____ Result ____/_____/____ Positive ( ) Negative ( )  Oropharynx (RT-PCR) Date ____/_____/____ Result ____/_____/____ Positive ( ) Negative ( )  Serology. Date ____/_____/____ (value) IgG ( ) IgM ( ) IgA ( )  Serology. Date ____/_____/____ (value) IgG ( ) IgM ( ) IgA ( )  Serology. Date ____/_____/____ (value) IgG ( ) IgM ( ) IgA ( )  Serology. Date ____/_____/____ (value) IgG ( ) IgM ( ) IgA ( ) | | | | | | | | | |
| **Prognostic tests for flu syndrome** | | | | | | | | | |
|  | Date | | Results | | Date | Results | Date | | Results |
| Lymphocytopenia (<1200) |  | |  | |  |  |  | |  |
| Thrombocytopenia |  | |  | |  |  |  | |  |
| Associated leucocytosis/neutrophilia |  | |  | |  |  |  | |  |
| Increase in DHL |  | |  | |  |  |  | |  |
| Increase in AST |  | |  | |  |  |  | |  |
| Increase in ALT |  | |  | |  |  |  | |  |
| Increase in D-dimer |  | |  | |  |  |  | |  |
| Increased ferritin |  | |  | |  |  |  | |  |
| Chest CT scan (the worst). Date: / / . Lung involvement: % | | | | | | | | | |
| Chest X-ray. Date: / / . Description: | | | | | | | | | |
| **Specific treatment for COVID-19 (enter the number of days of use)** | | | | | | | | | |
| Antibiotics: | | | | | | | | | |
| ( ) Hydroxychloroquine (days: ). Start: / / . ( ) Chloroquine (days: ). Start: / /  ( ) Azithromycin (days: ). Start: / / . ( ) Ivermectin (days: ). Start: / /  ( ) Annita (nitazoxamide). (days: ). Start: / / ( ) Ceftriaxone. (days: ). Start: / /  ( ) Other. _________________________________________________________ (days: ). Start: / / . | | | | | | | | | |
| Corticosteroids: | | | | | | | | | |
| ( ) Dexamethasone (days: ). Start: / / . ( ) Hydrocortisone (days: ). Start: / /  Methylprednisolone (days: ). Start: / / . | | | | | | | | | |
| Other: | | | | | | | | | |
| ( ) Oseltamivir (days: ). Start: / / . ( ) Enoxiparin (days: ). Start: / / .  ( ) Unfractionated Heparin (days: ) (daily dose: IU). Start: / / .  ( ) Other. _________________________________________________________ (days: ). Start: / / . | | | | | | | | | |
| Clinical classification of flu syndrome | | | | | | | | | |
| ( ) Asymptomatic ( ) Mild to moderate clinical illness ( ) Severe illness ( ) Critical illness  ( ) Death. Date: / / ( ) Critical unit ( ) Ward ( ) PA ( ) Home  ( ) Palliative care. Date: / / .Reason: ____________________________________________ | | | | | | | | | |
| Complications resulting from flu-like illness: ( ) No ( ) Yes. Which ones?  ( ) Sepsis ( ) Septic shock ( ) Arrhythmias ( ) Secondary infection ( ) Acute heart failure  ( ) Stroke ( ) AMI ( ) Thrombosis ( ) Other: ______________________________________ | | | | | | | | | |
| **NEWBORN** | | | | | | | | | |
| **Birth** | | | | | | | | | |
| Date: _____/____ /____ . Time:____:_____. Location: ( ) Hospital ( ) Home ( ) Other. What is it? ___________  Sex: ( ) Male ( ) Female Twin: ( ) No ( ) Yes  Type of delivery: ( ) Vaginal / Forceps ( ) Caesarean section. Reason ? ____________ Bolsa rota: ________ hours  Birth data: Weight___________g Length: ____________ cm Cranial circumference: __________cm  Gestational age (weeks): _____________ ( ) LMP ( ) US 1st trimester ( ) Capurro / New Ballard  Apgar: 1st minute ( ) 5th ( ) 10th ( )  Delivery room resuscitation: ( ) No ( ) Yes PPV CPAP IOT Cord catheterisation Massage  Maternal care (skin-to-skin contact) ( ) Yes ( ) No  Skin-to-skin contact 1st hour of life: ( ) Yes ( ) No. Reason: ___________________________________  Breastfeeding in the first hour of life: ( ) Yes ( ) No. Reason: _____________________________________  Kept with mother (next door): ( ) Yes ( ) No. Reason: _______________________________________ | | | | | | | | | |
| **Neonatal Unit** | | | | | | | | | |
| RN referred to:  ( ) AC (with mum). Stay (days): ______________Cause: ___________________________  ( ) Nursery. Permanência (dias):_____________________Cause:________________________  ( ) Neonatal ICU. Permanência (dias):_____________________Cause:____________________  ( ) Exclusive breastfeeding ( ) Yes ( ) No. Reason: __________________________________  ( ) Did you receive other types of milk ( ) No ( ) Yes. Which ones? ____________________________  Symptoms/intercurrences: ( ) No ( ) Yes (put the day of life and give details if necessary).  ( ) Fever. ____________________________________________________________  ( ) Hypothermia. ________________________________________________________  ( ) Dyspnoea/tachypnoea. _________________________________________________  ( ) Tachycardia/arrhythmia. _________________________________________________  ( ) Cyanosis. __________________________________________________________  ( ) Jaundice. __________________________________________________________  ( ) Cholestasis. ________________________________________________________  ( ) Hypoglycaemia. ______________________________________________________  ( ) Hypokalaemia/hypocalcaemia. ____________________________________________  ( ) Meningitis/convulsion. _________________________________________________  ( ) Irritability. ________________________________________________________  ( ) Diarrhoea. ____________________________________________________________  ( ) Difficulty breastfeeding. _______________________________________________  ( ) Urinary alteration. ____________________________________________________  ( ) Low weight gain. __________________________________________  ( ) Other: _____________________________________________________________  ( ) Other: _____________________________________________________________  ( ) Other: _____________________________________________________________  ( ) Other: _____________________________________________________________  ( ) Other: _____________________________________________________________ | | | | | | | | | |
| **Discharge of the newborn from hospital** | | | | | | | | | |
| Date: _____/____ /____ Weight: _____________  Newborn screening: ( ) Ear prick ( ) Heart prick ( ) Tongue prick ( ) Peek test  Discharged on exclusive breastfeeding: ( ) Yes ( ) No. Reason: _________________________  Medicines on discharge: ______________ ________________ ________________ ____________________  Diagnosis of the newborn at discharge:  1. ______________ 1. ______________ 1. ______________ 1. ______________ 1. ______________  1. ______________ 1. ______________ 1. ______________ 1. ______________ 1. ______________  1. ______________ 1. ______________ 1. ______________ 1. ______________ 1. ______________ | | | | | | | | | |
| **LABORATORY / SUBSIDIARY TESTS** | | | | | | | | | |
| Pregnant/Nursing | | | | NB/infant | | | | | |
| Pregnancy (SARS) | | Date: | |  | | | |  | |
| RT-PCR | |  | |  | | | |  | |
| IgA Sars-CoV-2 (blood) | |  | |  | | | |  | |
| IgG Sars-CoV-2 (blood) | |  | |  | | | |  | |
| IgM Sars-CoV-2 (blood) | |  | |  | | | |  | |
